# The peer review game: an agent-based model of scientists facing resource constraints and institutional pressures

**DOI:** 10.1007/s11192-018-2825-4

**Published:** 2018-07-09

**Authors:** Federico Bianchi, Francisco Grimaldo, Giangiacomo Bravo, Flaminio Squazzoni

**Affiliations:** 1 Department of Economics and Management, University of Brescia, Via San Faustino, 74/B, 25122 Brescia, Italy; 2 Department of Computer Science, University of Valencia, Avinguda de la Universitat s/n, 46100 Burjassot, Spain; 3 Department of Social Studies and Center for Data Intensive Sciences and Applications, Linnaeus University, Universitetsplatsen, 1, 35195 Växjo, Sweden

**Keywords:** Peer review, Cooperation, Game theory, Scientist strategies, Agent-based model

## Abstract

This paper looks at peer review as a cooperation dilemma through a game-theory framework. We built an agent-based model to estimate how much the quality of peer review is influenced by different resource allocation strategies followed by scientists dealing with multiple tasks, i.e., publishing and reviewing. We assumed that scientists were sensitive to acceptance or rejection of their manuscripts and the fairness of peer review to which they were exposed before reviewing. We also assumed that they could be realistic or excessively over-confident about the quality of their manuscripts when reviewing. Furthermore, we assumed they could be sensitive to competitive pressures provided by the institutional context in which they were embedded. Results showed that the bias and quality of publications greatly depend on reviewer motivations but also that context pressures can have a negative effect. However, while excessive competition can be detrimental to minimising publication bias, a certain level of competition is instrumental to ensure the high quality of publication especially when scientists accept reviewing for reciprocity motives.

## Introduction

Today, science is characterised by a “publish or perish” mentality due to growing competition for funds and academic job uncertainty (Edwards and Siddhartha 2017; Grimes et al. 2018). This context can undermine the objectivity and integrity of research as scientists could be induced to produce “publishable” results at all costs, thereby also putting journal editors and reviewers in a difficult position (Fanelli 2010; Marušić et al. 2013; Tijdink et al. 2014; D’Andrea and O’Dwyer 2017; Sarigöl et al. 2017; Bravo et al. 2018). Furthermore, the lack of robust positive and negative incentives for peer review (Hauser and Fehr 2007; Aktipis and Thompson-Schill 2010; Squazzoni et al. 2013) makes cooperation between editors, reviewers and authors extremely sensitive to external institutional conditions, e.g., competition pressures. Such a context seems to conspire against the quality and sustainability of peer review as rewards are for publishing, not for reviewing (Tennant et al. 2017).

In an influential contribution, Merton (2000 [1942]) suggested that science developed an institutional system that socialised scientists as members of a community towards robust and socially shared standards of conduct, social norms and values. He suggested that these ethical standards were intrinsic to the very idea of a scientific community in the modern sense of the word. He claimed that, although potentially ambiguous, these standards were instrumental to induce scientists to maintain the quality of science as a public good by means of a complex balance between incentives to collaborate and incentives to compete for recognition and prestige. He also suggested that the social organisation of scientific inquiry was context-dependent given that as science became more institutionalized, it also became “more intimately interrelated with the other institutions of society” (Merton 2000 [1942], p. 200). While discussing the diversity of scientist motivations, he suggested that:

assumption of a single motive is of course unsound. [...] Scientific inquiry, like human action generally, stems from a variety and amalgam of motives in which the passion for creating new knowledge is supported by the passion for recognition by peers and the derivate competition for place [...]. Any extrinsic rewards – fame, money, position – is morally ambiguous and potentially subversive of culturally esteemed values. [...] An excess of incentives can produce distracting conflict. But when the institution of science works effectively (and, like other social institutions, it does not always do so), recognition and esteem accrue to those scientists who have best fulfilled their roles, to those who have made fundamental contributions to the common stock of knowledge. (Merton 2000 [1942], p. 218)

Just as science was interrelated “with the other institutions of society” in the Mertonian era, in which government agencies were developing big science programmes, public funds were generous and academic institutions were expanding, so is science interrelated to institutions today. Recent research suggests that the current institutional incentive structure tends to trigger competitive spirits of scientists and pay-off preferably certain activities (e.g., publications, citations and grants). This implies that scientists need to strategise resource allocation (Fang and Casadevall 2015). Strategising here means rationally allocating scarce resources towards more rewarding activities, e.g., publications, grant proposals or lobbying for academic career. This means disinvesting to less rewarding ones, e.g., reviewing (Righi and Takács 2017).

Therefore, peer review can be influenced by a variety of exogenous change pressures, including technology and political demands of public accountability (see Peres-Neto 2016; Csiszar 2016). Furthermore, normative tension can occur between the multifaceted priorities of individual scientists (i.e., high publication and citation scores, big grants, consideration and peer esteem) and the real priority of the scientific community, i.e., scientific knowledge development. Although intrinsic to scientific community since the Mertonian era, this misalignment between individual priorities and collective interests has intensified today due to institutional pressure on competition and increasing uncertainty for funding and careers (Balietti et al. 2016).

It is worth noting that social dilemmas, where pay-offs of individuals are at odds with collective achievements, has been examined comprehensively by game theory-influenced experimental and behavioural science (see Fehr and Gintis 2007; Bravo and Squazzoni 2013). Research suggests that the alignment of individual and collective interests is possible whenever certain informal or formal enforcement options have been developed, such as positive incentives (e.g., rewards), negative incentives (social or institutional punishment), or when formal organisations or bureaucracy exist that help to coordinate individual behaviour towards socially desirable outcomes (Bowles 2016).

The problem here is that these mechanisms either do not exist or are weakly present in peer review. Peer review is a form of volunteer cooperation that involves academic community members who are called on to decide whether to contribute to a public good, while simultaneously having different priorities and pressures (see Northcraft and Tenbrunsel 2011). These priorities can determine the type of effort scientists eventually decide to allocate to reviewing, which might even vary from time to time and from scientist to scientist (see Leek et al. 2011).

Unfortunately, a few previous studies have seriously looked at peer review as a cooperation dilemma with experimental protocols and models that were inspired by game theory. For instance, Leek et al. (2011) performed an online game experiment on author-reviewer cooperation in open and closed peer review. The results showed that when reviewer behaviour was made public under open review, cooperation significantly increased as reviewers could be rewarded for reviewing. Squazzoni et al. (2013) designed an adapted version of a repeated investment game by adding reviewers who rated authors (trustees) to benefit editors (investors) and manipulated reviewer incentives. They found that cooperation increased and fairness equilibria were more likely to be achieved when reviewers were not materially incentivised. García et al. (2015c) proposed an adverse selection model based on agency theory to examine strategic interaction between journal editors and reviewers according to which reviewers were seen as “agents” of journal editors (their “principals”). They tested the implications of unobservability of reviewer’s expertise from the editor’s perspective and the ambiguity of review’s complexity, which can be precisely estimated only by the reviewer. Their simulation results suggested that bias was reduced when reputational rewards for reviewers were established as this stimulated talented reviewers to contribute, thereby helping editors in matching the complexity of manuscripts and the reviewers’ expertise more efficiently. Similarly, García et al. (2015a) looked at the relationship between reviewers’ effort and bias to understand optimal journal strategies of associate editor assignment and reviewer selection that could reduce potential bias (see also Cabotà et al. 2014), while García et al. (2015b) examined the potentially positive role of editor bias as a signal to match authors and journal of similar quality standards and so create heterogeneity of outlets.

These contributions were mainly concerned with identifying strategies and policies that could promote cooperation between editors and reviewers, beneficial to the quality of science as a public good (Righi and Takács 2017). Here, on the other hand, we aimed at looking in detail at the conflict between different options that scientists could take to solve an individual trade-off problem, i.e., how much resources they should allocate to multiple tasks under scarcity constraints. Furthermore, we were not concerned with finding optimal solutions to the game. Rather, we wanted to understand potential implications of scientist behaviour on aggregate system’s behaviour.

We first looked at an iterated version of a cooperation game between authors and reviewers in which, for the sake of simplicity, editors were synthesised in a final editorial decision of manuscripts’ acceptance or rejection, which was based only on reviewer opinion. We hypothesised that scientists could react adaptively to circumstances (i.e., publication success or failure) and estimate the fairness of peer review to which they were previously exposed. We also hypothesised that scientists could be realistic or excessively over-confident on the quality of their manuscripts. Finally, we tested the impact of competitive pressure from the institutional context. Our manipulations were intended to estimate the impact of these behavioural factors on the quality and efficiency of peer review, which were measured in terms of publication bias, wasted author investment and reviewing expense at a system level, following previous work by Squazzoni and Gandelli (2012, 2013), Cabotà et al. (2013) and Bianchi and Squazzoni (2016). In order to add behavioural realism to the model, we implemented a game-theoretic framework in an agent-based model (ABM), which allowed us to understand the sensitivity of aggregate outcomes to variation and heterogeneity of scientist behaviour (Thurner and Hanel 2011; Allesina 2012; Squazzoni and Gandelli 2012; Cabotà et al. 2013; Paolucci and Grimaldo 2014; Kovanis et al. 2016, 2017; Righi and Takács 2017).

The rest of the paper is organised as follows. “The PRG model” section presents the peer review game (PRG, from now on) model and briefly estimates certain analytical predictions on a simple version of the game. “The PRG agent-based model” section illustrates the set of scientist strategies that we tested with an ABM of the game. It is worth noting that while analytic game predictions can fully inform us about expected outcomes of peer review if scientists were all fully rational and self-interested, simulating the game with complex combinations of scientist behaviour and interaction effects was pivotal in understanding different aggregate outcomes (see Squazzoni and Takàcs 2011). “Results” section presents our simulation results, while “Discussion and conclusions” section summarises our key findings and discusses limitations and possible developments of our work.

## The PRG model

### Model overview

The PRG is a repeated game where *n* scientists were assigned the task of producing manuscripts for publication and reviewing. At each repetition, each scientist *i* played the game as author and referee simultaneously, by submitting a manuscript and reviewing another one. Each review provided a recommendation based on the estimated quality of the manuscript.

In order to perform their tasks, scientists were endowed with a given amount of resources $R_i$. Although a variety of resources are typically needed to perform these tasks (e.g., laboratory equipment, a team of collaborators, etc.), for the sake of simplicity, we assumed that $R_i$ was the time that each scientist *i* decided to devote to these tasks in a given period, say a month. We assumed that a fixed overall amount of resource $R = \sum _{i=1}^n R_i$ was available in the scientific community and that *R* was shared among all scientists following an arbitrary distribution.

Before each game, scientists had to decide how much resources to allocate to (1) produce manuscripts and (2) provide reviews of others according to a trade-off that mimicked availability of time as a given constraint. The allocation choice of scientists depended on a parameter that defined their submission effort $e_i \in [0,1]$, which represented the share of resources used by each scientist to produce a manuscript. The quality of each scientist *i*’s manuscript depended on their level of resources and effort:

1 $$\begin{aligned} Q^s_i = e_i R_ i \end{aligned}$$

while the quality of their review depended on the amount of resources that was left after producing the manuscript:

2 $$\begin{aligned} Q^r_i = R_i - Q^s_i = (1 - e_i) \, R_i. \end{aligned}$$

Following Squazzoni and Gandelli (2012, 2013) and Bianchi and Squazzoni (2016), we assumed that reviews were always error-prone, which means that the actual quality of a manuscript could be only approximately recognised by a reviewer. The estimated quality of a manuscript was calculated as:

3 $$\begin{aligned} \hat{Q}_i^s = \alpha _j Q^s_i \end{aligned}$$

with $\alpha _j$ that was drawn from a normal distribution having $\mu = 1$ and $\sigma = T^* - \min (T^*, Q^r_j)$, where *j* was the reviewer and $T^*$ was a quality threshold which estimated the minimum amount of resources (i.e., time) needed by each *j* to provide a fair review, i.e., a review that reflected at best the true quality of the manuscript. This meant that the closer $Q^r_j$ was to $T^*$, the fairer the review was. In addition, the review acknowledged the true quality of the manuscript $\forall \, Q^r_j \ge T^*$. The $T^*$ parameter reflected the difficulty of the review process in terms for instance, of time needed to provide a fair review to the manuscript.

Once the estimated quality of all manuscripts was assigned by the reviewers, a fixed proportion *P* of manuscripts was selected for publication on its basis, i.e., following $\hat{Q}_i^s$ ranking. Finally, the publication record ($p_i$) of scientists whose manuscripts were published was increased by one unit.

Every *m* rounds of repetitions, resources were redistributed proportionally to the scientists’ publication record. The new $R_i$ was calculated as *R* by the ratio between the size of *i*’s publication record and the total number of manuscripts produced by all scientists in the game until the current round as follows:

4 $$\begin{aligned} R_i = \frac{p_i}{\sum _{i=1}^n p_i} \, R. \end{aligned}$$

After resources were redistributed, a new round of the game started that used updated $R_i$ and $p_i$ values.

The overall rationale of the model was that scientists competed for publication but were also members of public research agencies or universities, which could not fire them. Considering that time was equal to resources, Eq. 4 implied that successful scientists were likely to devote even more time to instrumental scientific activities, e.g., preparation of grant proposals and management of large scientific teams. On the other hand, unsuccessful scientists could end up with a $R_i$ value that was close to zero and they were likely to allocate more time to teaching or administrative tasks.

### Equilibrium dynamics

We first studied the PRG analytically in a simplified $2 \times 2$ version. Here, we assumed that the game was played by two scientists who could choose between high or low effort in preparing manuscripts to submit $\{H_s,L_s\}$ and reviewing $\{H_r, L_r\}$. Being the reviewing effort $1 - e_i$, scientists could not simultaneously play $H_s$ and $H_r$. This meant that only two possible strategies were possible, i.e., $(H_s, L_r)$ and $(L_s, H_r)$. We assumed that, if a reviewer played high effort in reviewing (i.e., $H_r$), a high effort by the author to prepare the manuscript ($H_s$) always led to the publication of the manuscript (probability of publication $p = 1$), while an author’s low effort strategy to prepare the manuscript ($L_s$) always led to manuscript rejection ($p = 0$). If a reviewer played a low effort strategy ($L_r$)—e.g., by providing excessively positive, or negative reviews or not so informative reports—the acceptance of the manuscript was determined by chance alone, with $p = 0.5$ independently of the author’s strategy. This led to a matrix payoff as in Table 1. Note that payoffs for the referees were always equal to zero, as we assumed that reviewing did not increase the change of publication.

**Table 1:**
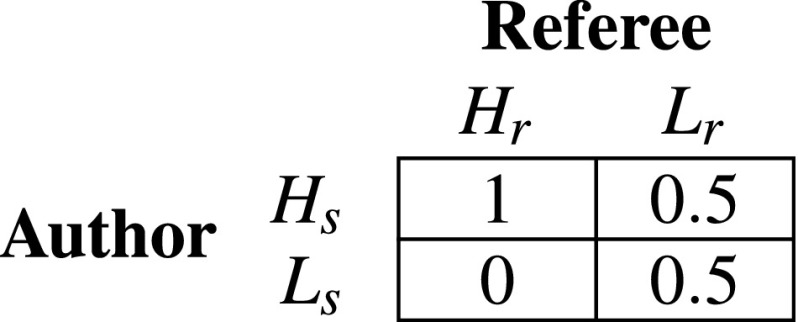
Authors’ payoffs in the simplified $2 \times 2$ PRG

Payoffs are expressed as probability of acceptance of the manuscript

Given that in each round players played both authors and referees and given the limitations in the choice of strategies discussed above, the combined payoff matrix takes the form showed in Table 2.

**Table 2:**
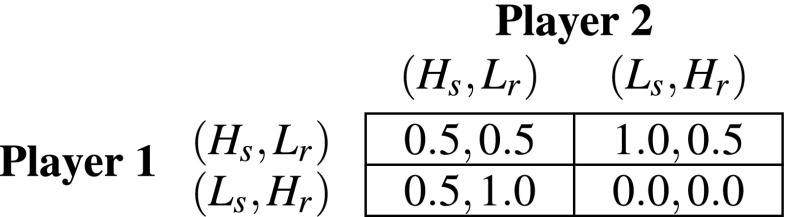
Combined payoff matrix in the $2 \times 2$ PRG

In each round, players play as both authors and referees

This situation did not change even if the number of scientists were higher or continuous effort choices were introduced. In general, the probability of publication for each scientist $i \in \{1, \ldots , n\}$ was an increasing function of the individual submission effort $e_i$ but a decreasing one of the average effort of all other scientists $\bar{e} = (\sum _{j \ne i} e_j) / (n - 1)$. This was because $Q_i^s$ increased with $e_i$ but there was an inverse relation between the amount of resources that were allocated to produce and submit papers and the randomness in the publication selection process.

Nevertheless, at least for low $T^*$ values, unfair equilibria could be reduced such that some scientists could accept a minimal amount of unfairness by holding a $e_i$ small enough to guarantee $Q^r_i \ge T^*$ in a situation where others invested all their resources to try to be published. This meant that unfair equilibria could be less robust here than in the $2 \times 2$ game. Another possible solution to the dilemma is that scientists with very high $R_i$ could invest a small part of their resources to provide $Q^r_i \ge T^*$, as this would not significantly decrease the probability that their manuscripts would be published. In these “Olsonian” (Olson 1965) cases, a public-good fair peer review could be at least partially provided even assuming that scientists were all similarly fully rational, i.e., selfishly maximising their chances of being published.

## The PRG agent-based model

In this extended version of the game,^1^ we hypothesised various behavioural strategies by scientists and manipulated the institutional setting in which they were embedded. For “behavioural strategies”, we meant resource allocation decision rules that the scientists could follow while managing the trade-off between investing in their manuscripts or reviewing. While considering simple behavioural rules as a limitation, it is actually questionable whether scientists, like all humans, would be able to compute complex game equilibria in a situation of repeated games with multiple players. Here, following behavioural game theory, we used a game-theory framework less deductively and more inductively. By “institutional setting”, we meant a particular set of incentives provided by science policies or market forces, which frame and constrain scientist behaviour (Squazzoni et al. 2013).

The first sub-section presents a set of adaptive strategies of resource allocation that considered a mix of motivations. For instance, scientists may maximise their chances of being published but could also accept reviewing to intentionally maintain robust quality standards. Vice versa, they could accept reviewing more maliciously to use their position to penalise other manuscripts, even at their own expenses, e.g., time to read and review a manuscript. This is what has typically been observed in behavioural experiments (Fehr and Gintis 2007; Balietti et al. 2016).

In an initial set of scenarios, we assumed that scientists constantly revised their allocation decision by adaptively reacting to previous acceptance or rejection of their manuscripts. Indeed, being published or rejected in a round could induce scientists to invest more on the quality of their manuscripts to subsequently increase their publication chances. Furthermore, they could accept reviewing to reciprocate good reviews they received in the past as authors with good reviews in turn, or vice versa, to intentionally punish other scientists who they consider to be indirectly responsible for bad standards of peer review they were previously victims of (Squazzoni and Gandelli 2013).

In a second set of scenarios, we assumed that instead of simply reacting to previous publication success or failure, scientists could estimate whether they truly deserved to be accepted or rejected by comparing the quality of their manuscripts to the overall quality of published manuscripts. Here, previous simulation results showed that reciprocity strategies of scientists could reduce publication bias only when scientists considered the fairness of the process rather than the outcome (Squazzoni and Gandelli 2013).

In the last subsection, we tested the same strategies by assuming that scientists were exposed to an institutional context that triggered their “strive for excellence” competitive spirits. In this case, we hypothesised that scientists could estimate the quality of their manuscripts against the quality of the top published manuscripts as though they were pressured by academic institutions to get published only in top journals. Here, we also tested whether scientists, by being exposed to competitive pressures, could be subject to over-confidence and examined whether this was beneficial or detrimental to the quality of peer review and publication (Johnson and Fowler 2011).

### Scientist behavioural strategies

This section presents all the strategies followed by scientists in the PRG model. Each has been tested separately in different simulation scenarios. Under all strategies, the scientists varied the proportion of resources invested in either preparing their own manuscripts or reviewing other manuscripts by increasing or decreasing their investment in submission by a constant quantity $\varDelta e$. Note that scientists could not play high-effort strategies in both roles simultaneously, as resource allocation decision was constrained by scarcity (Eqs. 1, 2).

#### The selfish scientist

Behavioural findings suggested that selfishness does not dominate completely in any human society, otherwise we would have not seen all the complex forms of cooperation that have been developed over time, including peer review. However, selfishness is part of human nature and must be contemplated even when examining scientist behaviour. Recent cases of misconduct and fraud in science indicate that under certain conditions, scientists could also behave rationally and selfishly to the detriment of others, including the public image and prestige of the scientific community (Bar-Ilan and Halevi 2017). At each time step *t*, we assumed that scientists selfishly maximised their probability of being published by increasing their $e_i$ up to 1, independently of previous publication success or failure at step $t-1$. This strategy follows the dominant strategy of the simplified PRG (Table 1) and was used as a baseline to examine the following.

#### The equaliser scientist

Here, we assumed that scientists systematically allocated their resources preferably towards preparing manuscripts even when they were previously published. However, when previously rejected, they decreased their $e_i$ to invest more in reviewing submissions by other scientists. Indeed, scientists can react to publication failure by both attempting to punish other scientists through detailed reviews in case they deserve to be rejected or to promote others who deserve it, as a means of making peer review work better.

#### The reciprocating scientist

Here, we assumed that scientists were interested to have their manuscripts published but also had an intrinsic interest in contributing to the quality of peer review by providing accurate reviews. Behavioural experiments suggest that in many situations, individuals are willing to reduce their individual pay-offs to benefit others as a way either to reciprocate good behaviour or to punish wrongdoers (Fehr and Schmidt 2006). Therefore, we assumed that scientists invested resources on submissions until they had their manuscript published. They then reciprocated their success by reviewing other submissions. This meant that in each time step *t*, agents increased their level of $e_i$ if their submission at $t-1$ was published, otherwise they decreased $e_i$.

Note that no pure altruist strategy (i.e., agents always decreasing their submission effort) was tested, because empirical evidence suggests that any form of altruism that is not sensitive to concrete conditions—e.g., other behaviour—is rare. In general, evolutionary studies show that the infrequency of this type of behaviour was key for the emergence of cooperation. Indeed, only the presence of forms of conditional or strong cooperation has helped reduce the proliferation of selfish behaviour and so create conditions for social order, including robust norms and institutions (Gintis 2009; Bowles 2016). However, it may be the case of certain scientists who are not pressured by any scientific performance indicators, as being evaluated mostly on other issues (e.g., teaching or administrative duties), who intensely invest resources on reviewing also to receive updated information on scientific progress.

### Institutional settings

In a baseline scenario, we assumed that agents were not embedded in an institutional setting which exposed them to certain rewards and influences. However, scientists are embedded in such contexts in that their perceptions and behaviour could reflect certain characteristics of the environment, e.g., competition, rankings and high-quality publication standards. It is also likely that these forces reverberate on peer review, which is not an institutionally isolated mechanism (Squazzoni et al. 2013; Kovanis et al. 2016; Tennant et al. 2017).

Here, we tested *equaliser* and *reciprocating* scientists to find whether they could react not only to previous publication success or failure but also to the fairness of the peer review process. We assumed that scientists correctly estimated the quality of their manuscripts and the reviewer opinion by comparing the quality of their submission ($Q^s_i$) with the value of *C*. This parameter was set to a threshold value of a percentile of the distribution of $Q^s$ between published manuscripts. Scientists perceived that reviewers were fair if $Q^s_i \ge C$ in case of publication of their submission or if $Q^s_i<C$ in case of rejection.

We then assumed that *equaliser* scientists increased their investment in submissions if they perceived to be treated fairly by reviewers, as we assumed their confidence in the process to be reinforced as long as they perceived that peer review worked correctly. Otherwise, they relaxed their effort in publishing and increased their investment on reviewing.

Finally, *reciprocating* scientists reacted to the perceived fairness of the peer review system by increasing their effort in reviewing. They therefore decreased their $e_i$ only if they were evaluated fairly, while they increased it to react to the low quality of peer review by focusing on their own priorities.

#### “Strive for publication” versus “strive for excellence”

In order to test different kinds of institutional setting, we compared the effects of both *equalizer* and *reciprocating* strategies by changing the *C* threshold parameter value that agents used to evaluate the fairness of peer review. We assumed that agents could correctly assess the quality of each published paper and compare the quality of their own submission ($Q^s_i$) with either the third or the first quartile of the published papers. In the first case (*strive for publication*) agents used a relatively low standard as a proxy to assess whether their submission or rejection was fairly decided. On the contrary, in the latter case, the situation resembled a scenario where scientists *strive for excellence*.

#### Over-confidence bias

Furthermore, we assumed that scientists fell into an *over-confidence* trap in that they systematically overestimated the quality of their manuscripts when comparing the quality of other published manuscripts. It is worth noting that the over-confidence bias is predictably higher when individuals have high confidence of their own opinion, which may not be so rare among scientists who have invested years of research on a given theory or finding (see Pallier et al. 2002; Chambers and Windschitl 2004). However, given that scientists should be proof seekers, empiricist individuals in the first place, we assumed that they were only minimally over-confident in that they perceived that the quality of their manuscripts was only 10% higher than what it actually was.^2^

### Simulation design

We ran computer simulations of the model by combining different behavioural strategies and institutional settings on a total of 11 scenarios. Table 3 shows the model parameters. The $T^{*}$ parameter was set at 6, meaning that we assumed that performing a review with minimal required quality took 6 hours. While we chose not to report simulation outcomes with extreme $T^{*}$ values because they were not realistic, it is worth noting that these values ($T^{*} = \{ 4, 5, 7, 8\}$) did not generate significantly different results. However, the “Appendix” reports sensitivity analysis with other $T^{*}$ values. Finally, we also chose to run simulations by assuming uniform distributions of *R* and *e*. This was to focus our analysis on the effect of the manipulated behavioural and institutional settings. The “Appendix” reports robustness tests with various initial parameter distributions.

**Table 3:** Simulation parameters

Parameter	Value
*n*	500
Steps	500
Distribution of *e*	Uniform
Distribution of *R*	Uniform
$T^{*}$	6
$\varDelta e$	0.05
*P*	0.25

Following Squazzoni and Gandelli (2012, 2013) and Bianchi and Squazzoni (2016), we assessed the outcome of the simulated peer review process by measuring bias in manuscript selection and quality of published manuscripts.

At the end of each simulation run, $n * P = 125$ manuscripts were published. First, we calculated the *evaluation bias* as the percentage of rejected manuscripts among those 125 manuscripts with the highest $Q^s_i$ values, i.e. the proportion of incorrectly rejected manuscripts on the total amount of published articles. We then calculated the *publication quality* as the average $Q^s$ value among the published manuscripts. Finally, we estimated the *top quality* as the average $Q^s$ value among the top 10 published manuscripts ranked by $Q^s$.

## Results

Results were obtained by running 100 repetitions of the model for each combination of behavioural strategies and institutional settings. For each repetition, the model was run for 500 steps. At the end of each repetition, we calculated the cumulative moving average values of *evaluation bias*, *publication quality*, and *top quality* based on the last 100 runs. Finally, we averaged the results on the total number of repetitions.

**Fig. 1:**
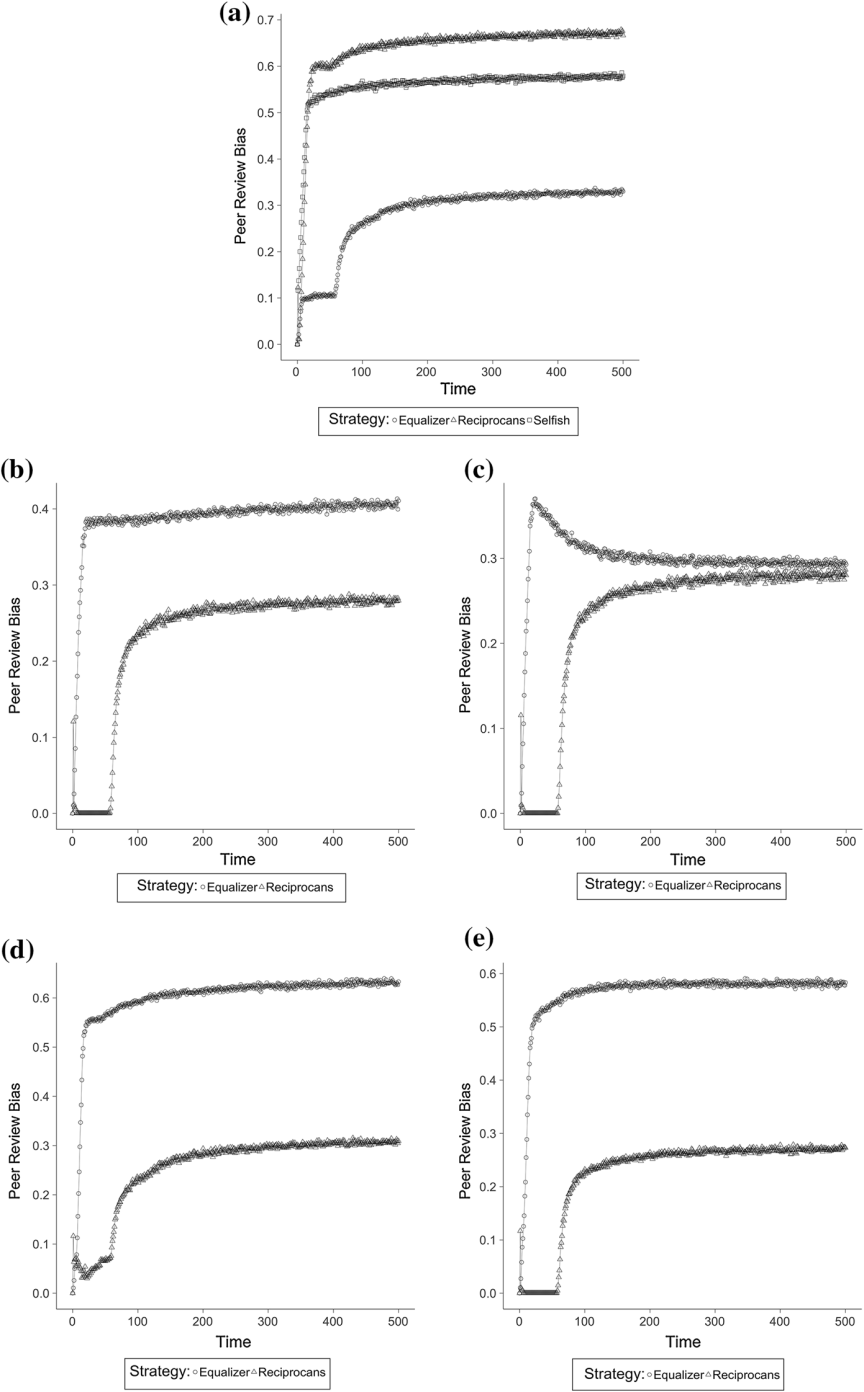
Evolution of *evaluation bias* over time for each institutional setting (results averaged over 100 repetitions for each scenario). **a** *No comparison*, **b** *strive for publication*—objective self-evaluation, **c** *strive for publication*—overconfident self-evaluation, **d** *strive for excellence*—objective self-evaluation, **e** *strive for excellence*—overconfident self-evaluation

Figure 1 shows average dynamics of *evaluation bias* with different behavioural strategies in each institutional setting. In each scenario, the system reached an equilibrium after nearly 350 runs.

Table 4 shows *evaluation bias* in each scenario. The second column (*no comparison*) shows different behavioural strategies when agents reacted only to the outcome of their past submissions. When scientists were selfishly interested only in their own publications’ success (*selfish*), 57.61% of papers of sufficient quality for publication were rejected. Therefore, in the baseline scenario (see “Scientist behavioural strategies” section) the allocation of manuscripts was approximately random. When we assumed that scientists increased their effort in reviewing only in case they were rejected as authors (*equaliser*), the average evaluation bias dropped to approximately one third of manuscripts. Otherwise, by assuming that scientists increased their reviewing effort in case they were previously published and decreased it otherwise, we obtained the worst result, with evaluation bias peaking at 66.91%.

**Table 4:** Average *evaluation bias* (%) in all simulation scenarios

Behavioural strategy	*No comparison*	Institutional setting			
		*Strive for publication*		*Strive for excellence*	
		Objective	Overconfidence	Objective	Overconfidence
*Selfish*	57.61				
*Equaliser*	32.71	40.56	29.47	62.79	58.01
*Reciprocating*	66.91	27.86	28.05	30.66	27.04

Tables 5 and 6 show the effect of behavioural strategies and institutional settings on the quality of publications. When considering *no comparison*, we found that *publication quality* and *top quality* were sensitive to behavioural strategies and *evaluation bias* (see Table 4). In general, by assuming that agents did not compare their own publication outcomes with others, the *equaliser* strategy generated the lowest bias and the highest overall quality. Figure 1 shows that the outcome was due to agents generally concentrating their resources preferentially on submissions. This produced relatively low levels of bias and high quality. However, this depended on the level of reviewing efforts: where these were low, bias stabilised around a fairly low level, so generating the second-highest *published quality* value across all scenarios. On the other hand, by assuming *reciprocating* strategies, agents decreased their submission efforts and this reflected on the average low quality of submissions, eventually making submissions more vulnerable to misjudgment due to reviewers’ perception bias. The symmetric increase in reviewing effort did not compensate low submission quality, so generating an equilibrium with high bias and low overall quality.

**Table 5:** Average publication quality in all simulation scenarios (normalized values ranging 0–1)

Behavioural strategy	*No comparison*	Institutional setting			
		*Strive for publication*		*Strive for excellence*	
		Objective	Overconfidence	Objective	Overconfidence
*Selfish*	0.60				
*Equaliser*	0.98	0.71	0.85	0.44	0.49
*Reciprocating*	0.41	0.00	0.01	1.00	0.36

**Table 6:** Average publication quality of top 10 published papers in all simulation scenarios (normalized values ranging 0–1)

Behavioural strategy	*No comparison*	Institutional setting			
		*Strive for publication*		*Strive for excellence*	
		Objective	Overconfidence	Objective	Overconfidence
*Selfish*	0.51				
*Equaliser*	0.91	0.94	1.00	0.75	0.83
*Reciprocating*	0.36	0.01	0.00	0.93	0.34

Results changed significantly when we assumed that agents adjusted their efforts on the basis of the average published papers’ quality (*strive for publication*). In this case, the effect of scientists’ strategies on *evaluation bias* was reversed. Bias generated by the *equaliser* strategy increased to 40.56%, while the assumption of a *reciprocating* strategy significantly decreased it, so generating the least overall bias in this setting (27.86%). However, the *equaliser* strategy produced a high *publication quality*—although less than in the previous setting—, while the *reciprocating* strategy provided the worst average quality across all scenarios. This was because *equaliser* agents were less likely to allocate resources on reviewing than in the *no comparison* setting. Low-quality reviews in turn, had a negative impact on the average quality of published papers, because a significant amount of high-quality papers were unfairly rejected. Interestingly, overall high submission efforts produced some very high-quality papers, which were not vulnerable to biased reviews. This explains why *top quality* increased. On the other hand, Fig. 1 shows that *reciprocating* agents’ tendency to allocate their efforts more fairly initially eliminated any bias. This later increased though minimally when biased rejections induced agents to decrease their reviewing efforts. Yet, overall unbalanced resource allocation on reviewing produced the worst average quality of published papers across all scenarios.

The assumption of agents’ *over-confidence* mitigated the impact of strategies on *evaluation bias* but generated significantly different quality outcomes. This was because *equaliser* agents were more likely to perceive that their rejected papers were higher quality than the published ones. Therefore, they started to devote more efforts on reviewing, which eventually lowered the overall bias. However, a small amount of agents rarely experienced rejections in the first period of the simulation, eventually achieving the highest levels of submission quality. This subset of agents generated the highest *top quality* result across all scenarios. This compensated other agents’ lower submission efforts and kept *publication quality* relatively high together with low *evaluation bias*.

A similar relationship between the two strategies can be observed in terms of *evaluation bias* when we assumed that scientists compared the publication outcome of their own submission with the average quality of the top 10 published papers (*strive for excellence*). Bias generated by both strategies increased, with *equaliser* strategy generating more than twice the bias of *reciprocating* strategy, rejecting on average 62.79% high-quality submissions. However, the relationship reversed if we consider results in *publication quality*. While this decreased among *equaliser* agents as compared to the other institutional settings (0.44), *reciprocating* agents performed the best results across all scenarios. This setting amplified the negative impact of *equaliser* strategies on the overall bias, because it made agents even less likely to react to an incorrect rejection by increasing their reviewing efforts than in the other settings. This yielded an overall high bias, eventually generating a drop in the average quality. Following these results, *top quality* was less affected by this negative effect. *Reciprocating* agents however were more likely to shift resources towards reviewing because they perceived they had deserved rejections. Moreover, some of them submitting high quality manuscripts could consider rejections as biased, so further increasing their efforts in submissions. This created an average high quality of published papers, among which top 10 scored one of the highest results across all scenarios (0.93). Interestingly, when we assumed *over-confidence*, the probability of perceiving a rejection as incorrect was so high that the overall high reviewing effort by *reciprocating* agents depressed *publication quality* (0.36).

## Discussion and conclusions

If we consider resource allocation strategies of scientists as if they were not embedded in any institutional setting, our results suggest that scientists could concentrate their time and efforts in performing research and competing for preparing high-quality submissions. Reviewing could be considered a side-activity for brilliant scientists, preferentially allocated to less successful scientists. If so, reviewing would not require a symmetrically high effort to compensate the lack of rigour of submitted manuscripts, thus determining a functional division of academic labour (Righi and Takács 2017).

However, considering that scientists are embedded in an institutional setting that determines priorities, norms and (positive/negative) incentives, such a division of labour can even be dysfunctional. For instance, when the institutional environment promotes scientific excellence (e.g., publishing in top journals in a stratified publication market), competition for publication could deteriorate resources allocated to reviewing to such a level as to impede peer review to recognise high-quality submissions. This could depress the average quality of published research (Righi and Takács 2017). In this case, a setting where not only top-tier publications are rewarded could allow scientists to pursue a more balanced resource allocation between publishing and reviewing (Squazzoni and Gandelli 2012, 2013; Bianchi and Squazzoni 2016). This would also make the emergence of an efficient self-organized “division of scientific labour” possible in that a high-class of top scientists could primarily concentrate their efforts on research, while a larger group would be more engaged in reviewing without depressing the average quality of publications (Kovanis et al. 2016).

Furthermore, our results suggest that an even better performance could be obtained if scientists internalised reciprocity norms that would signal reviewing as a means to indirectly reward previous cooperation by referees (Squazzoni et al. 2013). In this case, even a competitive focus on excellence triggered by the institutional context would find a sustainable equilibrium between bias minimisation, high-quality research and fairness of peer review. Here, even a certain amount of over-confidence could be instrumental in promoting high-quality publication. However, our simulations suggest that if competition were only mildly promoted, reciprocating scientists could disproportionately concentrate resources on reviewing, therefore generating a very fair system, yet selecting very low-quality scientific publications.

In conclusion, although highly abstract, our model helped us to understand the interplay of contexts and behaviour in peer review and explore different trade-offs in scientists’ resource allocation (Righi and Takács 2017). Obviously, empirical data would be necessary to verify our results. However, finding data that allow us to estimate these trade-offs at the individual level at a sufficient scale to map the process comprehensively is almost impossible. Finally, initiatives to improve reviewer recognition and rewarding more referees, on which there is vivid debate today, could help to promote more positive equilibria between research and reviewing and promote more shared reciprocity among scientists (Kovanis et al. 2017; Ross-Hellauer 2017; Tennant et al. 2017).

## Figures and Tables

**Fig. 2:**
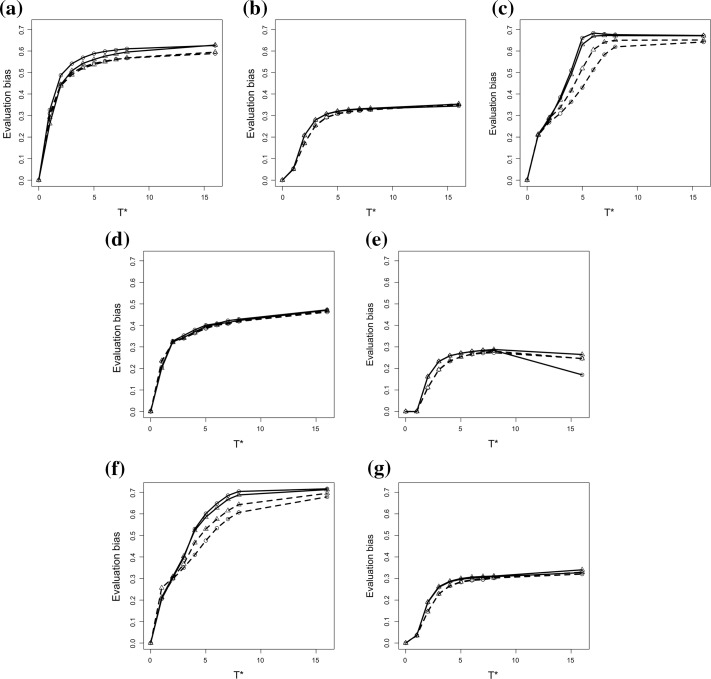
*Evaluation bias* across different $T^*$ values, initial resource distribution and initial effort distribution. **a** *Selfish*—*no comparison*, **b** *equaliser*—*no comparison*, **c** *reciprocating*—*no comparison*, **d** *equaliser*—*strive for publication*, **e** *reciprocating*—*strive for publication*, **f** *equaliser*—*strive for excellence*, **g** *reciprocating*—*strive for excellence*

**Fig. 3:**
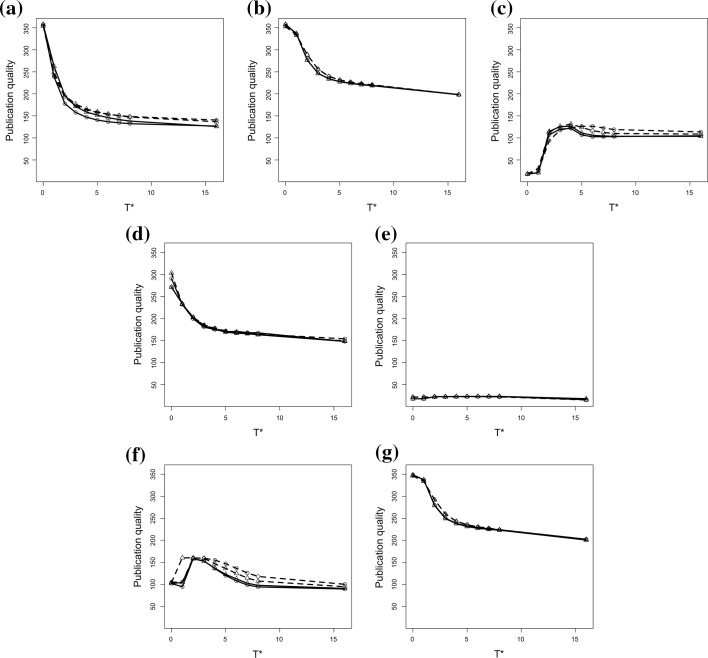
*Publication quality* across different $T^*$ values, initial resource distribution and initial effort distribution. **a** *Selfish*—*no comparison*, **b** *equaliser*—*no comparison*, **c** *reciprocating*—*no comparison*, **d** *equaliser*—*strive for publication*, **e** *reciprocating*—*strive for publication*, **f** *equaliser*—*strive for excellence*, **g** *reciprocating*—*strive for excellence*

**Fig. 4:**
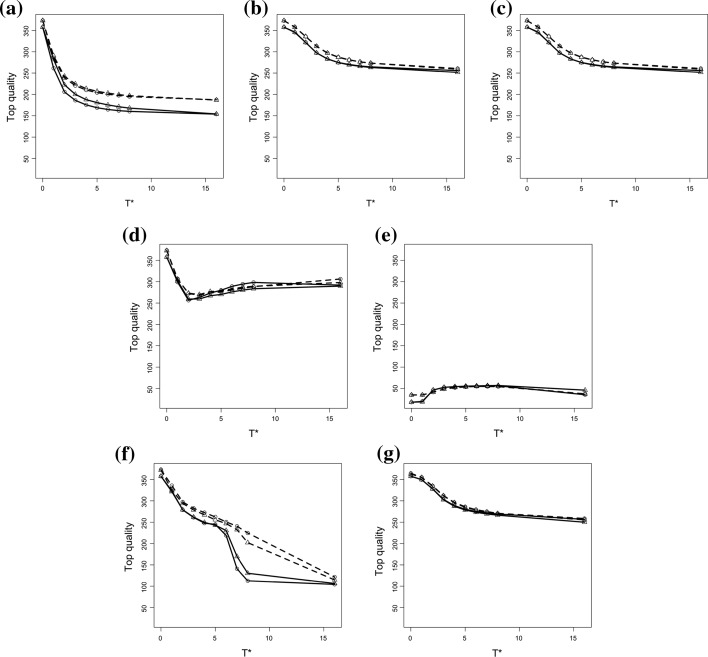
*Top quality* across different $T^*$ values, initial resource distribution and initial effort distribution. **a** *Selfish*—*no comparison*, **b** *equaliser*—*no comparison*, **c** *reciprocating*—*no comparison*, **d** *equaliser*—*strive for publication*, **e** *reciprocating*—*strive for publication*, **f** *equaliser*—*strive for excellence*, **g** *reciprocating*—*strive for excellence*

## Appendix

Figure 2 shows the sensitivity analysis of *evaluation bias* with different values of $T^*$. We also tested the robustness of our results while varying the initial distribution of $R_i$ and $e_i$.

Figure 3 shows the sensitivity analysis of *publication quality* with different values of $T^*$. We also tested the robustness of our results by varying the initial distribution of $R_i$ and $e_i$.

Figure 4 shows the sensitivity analysis of *top quality* with different values of $T^*$. We also tested the robustness of our results by varying the initial distribution of $R_i$ and $e_i$.

## Footnotes

1 A NetLogo (Wilensky 1999) version of the model can be accessed at https://www.comses.net/codebases/6b77a08b-7e60-4f47-9ebb-6a8a2e87f486/releases/1.0.0/.

2 Simulations with higher levels of the over-confidence rate did not yield significantly different results.
